# Concentration Dependence of the Antioxidant and Prooxidant Activity of Trolox in HeLa Cells: Involvement in the Induction of Apoptotic Volume Decrease

**DOI:** 10.3390/antiox9111058

**Published:** 2020-10-29

**Authors:** Maria Elena Giordano, Roberto Caricato, Maria Giulia Lionetto

**Affiliations:** Department of Biological and Environmental Sciences and Technologies (DiSTeBA), University of Salento, Via prov.le Lecce-Monteroni, 73100 Lecce, Italy; elena.giordano@unisalento.it (M.E.G.); roberto.caricato@unisalento.it (R.C.)

**Keywords:** Trolox, HeLa, antioxidant, prooxidant, AVD, apoptosis

## Abstract

Trolox (6-hydroxy-2,5,7,8-tetramethylchroman-2-carboxylic acid), a hydrophilic analog of vitamin E, is known for its strong antioxidant activity, being a high radical scavenger of peroxyl and alkoxyl radicals. Under particular conditions, Trolox may also exhibit prooxidant properties. The present work aimed at studying the dual antioxidant/prooxidant behavior of Trolox over a wide range of concentrations (from 2.5 to 160 µM) in HeLa cells. In particular, the study addressed the dose-dependent effects of Trolox on the oxidative cell status and vitality of HeLa cells, focusing on the potential role of the vitamin E analog in the induction of one of the first steps of the apoptotic process, Apoptotic Volume Decrease (AVD). In HeLa cells, Trolox showed significant antioxidant activity, expressed as the ability to reduce the endogenous ROS production detected by the ROS-sensitive probe 5-(and-6)-chloromethyl-2′,7′-dichlorodihydrofluorescein diacetate (CM-H_2_DCFDA), at low concentrations (range: 2.5–15 µM), but exerted a dose-dependent prooxidant effect at higher concentrations after 24 h exposure. The prooxidant effect was paralleled by the reduction in cell viability due to the induction of the apoptotic process. The dual behavior, antioxidant at lower concentrations and prooxidant at higher concentrations, was evident also earlier after 2 h incubation, and it was paralleled by the isotonic shrinkage of the cells, ascribed to AVD. The use of SITS, known Cl^−^ channel blocker, was able to completely inhibit the Trolox-induced isotonic cell shrinkage, demonstrating the involvement of the vitamin E analog in the alteration of cell volume homeostasis and, in turn, in the AVD induction. In conclusion, the study shed light on the concentration dependence of the Trolox antioxidant/prooxidant activity in HeLa cells and revealed its role in the induction of one of the first events of apoptosis, AVD, at high concentrations.

## 1. Introduction

Trolox (6-hydroxy-2,5,7,8-tetramethylchroman-2-carboxylic acid) is a hydrophilic analog of α-tocopherol, the most active and the most common form of tocopherols (vitamin E) in the human body. α-tocopherol is the major lipid antioxidant of biomembranes; it prevents membrane oxidative damage through inhibition of polyunsaturated fatty acids peroxidation by scavenging lipid peroxyl radicals [1,2]. Besides, it quenches and reacts with singlet oxygen and slowly reacts with superoxide anions [3]. Trolox shows the same antioxidant activity of α-tocopherol, but compared to α-tocopherol, which is lipid soluble, it lacks the phytyl tail, it is more hydrosoluble, and it has the advantage to reach both the water and the lipid compartments of cells.

Trolox is known for its high radical scavenging activity of peroxyl and alkoxyl radicals [4] and as such, it is often used as a reference in several biochemical assays, in which the radical scavenging activity of studied compounds is expressed as Trolox equivalents. It has been demonstrated to act as a strong antioxidant in several cellular model systems. In human skin fibroblasts, it was found to lower intracellular ROS levels and lipid peroxidation and to induce a less oxidized mitochondrial thiol redox state [5]. In IPEC-J2 cells, Trolox reduced intracellular oxidative stress, it improved wound-healing capacity and paracellular permeability following exposure to exogenous prooxidant compounds [6]. In thymocytes, it was able to prevent peroxynitrite-mediated oxidative stress and apoptosis [7]. Trolox contributed to the protection of human and murine primary alveolar type II cells from injury by cigarette smoke-induced oxidative stress [8] and prevented the generation of oxidative stress in primary adult rat optic nerve head astrocytes showing induced reactive astrocytosis [9]. Moreover, Trolox was demonstrated to prevent lipid peroxidation induced by CYP2E1 in Hep G2 cells [10], to attenuate impaired proliferation of oxidatively stressed myoblasts overexpressing parkin interacting substrate (PARIS/ZNF746) [11], to inhibit DNA damage formation induced by singlet oxygen in human lymphoblast WTK-1 cells27 [12], and to protect erythrocytes during photodynamic treatment [13].

Trolox has been reported to prevent oxidative stress-induced apoptosis in several cell types, such as mouse thymocytes [14], renal NRK-52e cells (normal rat kidney 52e) [15], rat myocardial H9c2 cells [16], renal proximal tubular epithelial LLC-PK1 cells [17].

Although Trolox possesses strong antioxidant activity, it might also exhibit prooxidant properties in particular conditions, as also observed for other antioxidant compounds, including α-tocopherol [18,19,20]. Like α-tocopherol, the Trolox antioxidant activity arises from its ability to donate hydrogen from the hydroxyl group of the chromanol ring [21] to reactive species. This, in turn, drives the formation of phenoxyl radical (PhO·), which can oxidize ascorbate and other biomolecules to radicals [21]. Phenoxyl radical can extract a hydrogen atom from the bisallylic methylene groups of polyunsaturated fatty acids, inducing lipid peroxidation, although the rate constant for this reaction is slow, being about 10^−1^ ± 0.05 M^1^s^1^ [22]. Several studies demonstrated Trolox to exhibit prooxidant properties in the presence of free metal ions [23]. For example, in astrocytes, Trolox increased the Cu^2+^-induced ROS generation and cytotoxicity [24], while in erythrocytes Trolox stimulated ferric ion-catalyzed ascorbate oxidation [25].

The synergic prooxidant action of Trolox has been described also for nonmetallic compounds. It has been reported to induce a synergistic prooxidant effect with superoxide generating selenium compounds in mouse keratinocytes [26], to potentiate As_2_O_3_-induced oxidative stress in APL cell line, myeloma, and breast cancer cells [27], and to enhance curcumin’s cytotoxicity in A2780 cells [28].

At higher concentrations, Trolox has been described to induce lipid peroxidation, GSH oxidation, and cytotoxicity [29,30].

The dual antioxidant/prooxidant behavior of Trolox is a multifaceted phenomenon, showing cell-type specificity and being influenced by the experimental conditions used. Concentration is one of the most determinant factors; however, most of the studies exploring the cellular effects of Trolox have been carried out on single concentrations of the vitamin E analog.

The present study aimed at investigating the dual antioxidant/prooxidant behavior of Trolox over a wide range of concentrations (from 2.5 to 160 µM) on a model cell line, HeLa cells. In particular, we addressed if there are dose-dependent effects of Trolox on the oxidative cell status of HeLa cells and if the vitamin E analog is involved in the induction of the apoptotic process focusing on one of the first step of the apoptotic process, Apoptotic Volume Decrease (AVD). One of the main hallmarks of apoptosis is represented by cell shrinkage, which occurs in two distinct stages: the early shrinkage, named, AVD, starting in the first hours, represented by an isotonic shrinkage of the cells [31,32,33], and the second phase related to cell fragmentation or formation of the apoptotic body [34]. Recent studies have pointed out the importance of AVD in the onset and progression of apoptosis, suggesting its critical role in the regulation of apoptotic nucleases and the activation of caspases. AVD arises from a net loss of K^+^, Cl^−^, and organic osmolytes from the cell [31,35,36], which, in turn, is followed by an osmotic loss of water and consequent cell shrinkage. In particular, in the present work, we addressed if Trolox can induce AVD thought its effect on Cl^−^ channels.

In this study, HeLa cells were chosen as the experimental cell model for addressing the objectives of the work. HeLa cells are one of the most studied cellular models, widely utilized in several fields from cancer research, to drug development, gene expression, and cell death pathways. Moreover, they are particularly useful for the objectives of this study because their AVD response is well known and characterized [37].

## 2. Methods

### 2.1. Materials

All chemicals were reagent grade. Cell culture materials were acquired from EuroClone (Paignton-Devon, UK). The cell-permeant probe 5-(and-6)-chloromethyl-2′,7′-dichlorodihydrofluorescein diacetate (CM-H_2_DCFDA) and Alexa Fluor^®^ 488 annexin V were purchased from Life Technologies-Molecular Probes (Waltham, MA, USA). All the other reagents were purchased from Sigma Aldrich (St. Louis, MO, USA). HeLa cells were purchased from ATCC (Manassas, VA, USA).

### 2.2. Intracellular Oxidative Stress Detection

The intracellular oxidative stress was assessed using a ROS-sensitive probe, 5-(and-6-)-chloromethyl-2′,7′-dichlorodihydrofluorescein diacetate, acetyl ester (CM-H_2_DCFDA) (Ex/Em: 492–495/517–527 nm) (Thermo Fisher Scientific, Waltham, MA, USA). HeLa cells were grown as a monolayer in Dulbecco’s Modified Eagle’s Medium with 4500 mg glucose/L (DMEM) supplemented with 10% FBS, 40 IU/mL penicillin G, 2 mM L-glutamine, and 100 µg/mL streptomycin under a 95% air/5% CO_2_ atmosphere. Cells were plated (1 × 10^5^ per mL) into 96-well black plate and incubated for 24 h to allow the cells to attach to the bottom of the plate. Afterwards, the cells were incubated with increasing concentrations of Trolox (respectively, 2.5, 5, 10, 15, 20, 40, 80, and 160 mM) for 2 or 24 h. Then, they were charged with the cell-permeant fluorescent probe CM-H_2_DCFDA 5 µM. Subsequent oxidation, this probe yields a fluorescent adduct that is trapped inside the cell. Fluorescence was then measured by the Synergy™ (BioTek Instruments, Inc., Winooski, VT, USA) multi-mode microplate reader.

### 2.3. Cell Viability Assessment by MTT Assay and Propidium Iodide

Cell viability was assessed by the MTT test according to Latronico et al. [38]. The MTT assay was carried out after plating HeLa cells (1 × 10^5^ per mL) into 96-well plate. After 24 h incubation, the cells were preincubated with Trolox at the final concentrations of 2.5, 5, 10, 15, 20, 40, 80, and 160 µM for 24 h. Then, 20 μL MTT (0.5 mg/mL in PBS) was put on each well. After an incubation of 4 h at 37 °C, the medium was discharged and DMSO (100 µL) was added. Absorbance was measured at 570 nm with a spectrophotometer (EON BioTek Instruments, Winooski, VT, USA).

For propidium iodide cell viability assessment, HeLa cells (1 × 10^5^ per mL) were plated into 6-well plate and incubated for 24 h. Then, the cells were incubated with Trolox (2.5, 5, 10, 15, 20, 40, 80, and 160 µM) for 24 h. Following incubation, they were washed with PBS, detached by gentle trypsinization, and then incubated with propidium iodide (final concentration of 50 µg/mL) for 10 min. Then, cells were washed again and spectrofluorimetrically analyzed by Synergy™ (BioTek Instruments, Inc., Winooski, VT, USA) multi-mode microplate reader (λ_ex_: 535 nm and λ_em_: 617 nm).

### 2.4. Estimation of Changes in Cell Volume

Cell volume changes were assessed by the measurement of changes in cell size through morphometric analysis of the cell area according to Lionetto et al. [32]. Cultured cells were observed by an inverted microscope in bright field (NIKON TE300 Eclipse E600, Nikon, Tokyo, Japan), and the 2-dimensional images obtained from a video camera (TK-C1381, JVC, Yokohama, Japan) were digitalized and analyzed using the LUCIA images analysis software (Nikon, Tokyo, Japan). At least a minimum of 300 cells/condition was analyzed.

### 2.5. Detection of Apoptosis

HeLa cells (1 × 10^5^ per mL) were plated into 6-well plate and incubated for 24 h. Then, the cells were preincubated with Trolox at the final concentrations of 2.5, 40, 80, and 160 µM for 14 h. Then, they were washed with PBS, detached by gentle trypsinization, and then resuspended in annexin V binding buffer (HEPES 10 mM, pH 7.4, NaCl 150 mM, CaCl_2_ 2.5 mM in PBS) and incubated with Alexa Fluor^®^ 488 annexin V (final concentration 2.5 µg/mL) and propidium iodide (final concentration 50 µg/mL) for 10 min. Then, the cells were washed and transferred in a 96-well black plate. Fluorescence was measured by the Synergy™ (Biotek) multi-mode microplate reader (annexin V: λ_ex_: 488 nm and λ_em_: 530 nm; propidium iodide: λ_ex_: 535 nm and λ_em_: 617 nm).

### 2.6. Statistics

Values are given as the mean ± S.E.M. The statistical significance of data was analyzed by one-way ANOVA, Dunnett’s test, and Student’s *t*-test. Percentage values were arcsin transformed before the analysis. Data are expressed as mean ± SEM.

## 3. Results

### 3.1. Dose-Dependent Effect of Trolox on Basal ROS Production and Cell Viability after 24 h Exposure

**Figure 1:**
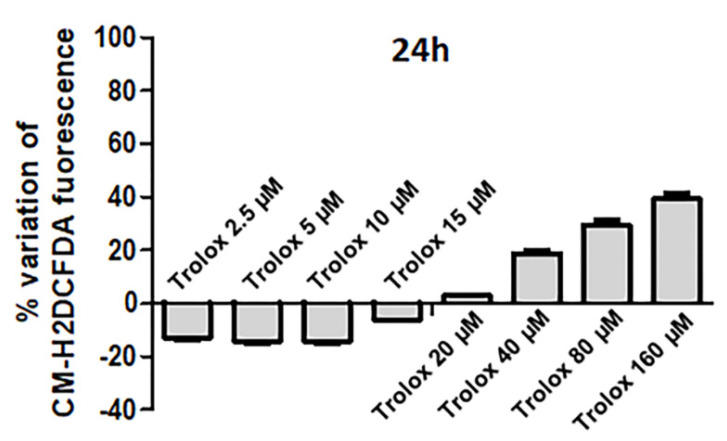
Percentage variation of fluorescence intensity in HeLa cells exposed for 24 h to increasing concentrations of Trolox (from 2.5 to 160 µM) and then charged with 5-(and-6)-chloromethyl-2′,7′-dichlorodihydrofluorescein diacetate (CM-H_2_DCFDA). The ordinates indicate the percentage variation of the probe fluorescence intensity, which was calculated as follows: (fluorescence of control cells − fluorescence of treated cells)/(fluorescence of control cells) × 100. Data are expressed as mean ± SEM of 3 independent experiments.

HeLa cells were treated for 24 h with increasing concentrations of Trolox in the range of 2.5–160 µM, and then they were charged with the cell-permeable ROS-sensitive probe CM-H_2_DCFDA to investigate the effect of the vitamin E analog on the intracellular oxidative status of the cells. The incubation time of 24 h was established based on previous works, demonstrating 24 h incubation as an appropriate time period for Trolox-induced biological responses to be evoked in several cell types [39,40,41]. Figure 1 shows the percentage variation of the fluorescence emitted by the cells calculated compared to control cells (not exposed to Trolox). Trolox was able to exert an antioxidant activity at lower concentrations (2.5–15 µM), as indicated by the negative percentage variation of the CM-H_2_DCFDA fluorescence, which is indicative of a decrease in basal ROS production. The antioxidant effect size was about 20% in the concentration range of 2.5–10 µM. A decrease in the antioxidant effect was recorded at 15 µM Trolox. The antioxidant activity disappeared at 20 µM. On the other hand, at higher concentrations (from 40 to 160 µM) a dose-dependent prooxidant effect was recorded, as indicated by the positive increase in the percentage variation of the CM-H_2_DCFDA fluorescence.

These results demonstrate a dose-dependent dual behavior of Trolox on the basal ROS production of HeLa cells following 24 h exposure.

**Figure 2:**
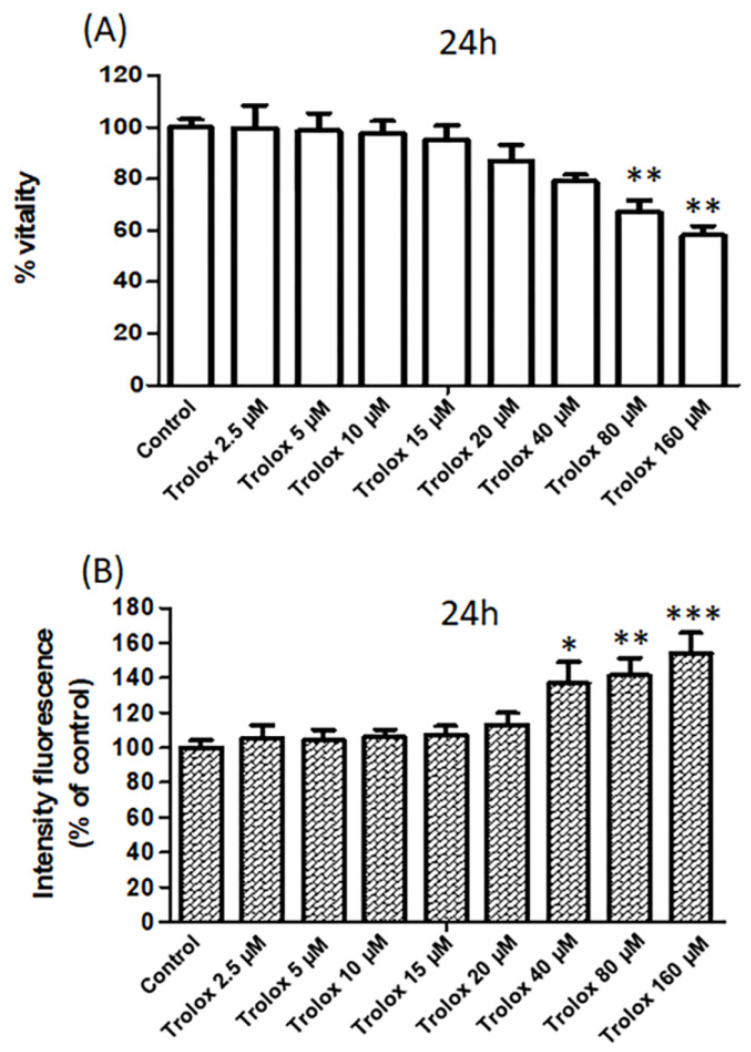
(**A**) The vitality of HeLa cells exposed for 24 h to increasing concentrations of Trolox (from 2.5 to 160 µM) assessed by MTT test. Data are expressed as percentage vs. control. (**B**) The vitality of HeLa cells exposed for 24 h to increasing concentrations of Trolox (from 2.5 to 160 µM) assessed by the fluorescence intensity (% vs. control) of propidium iodide. Data are expressed as mean ± SEM of 3 independent experiments. The statistical significance of data was analyzed by one-way ANOVA and Dunnett test. * = *p* < 0.05; ** = *p* < 0.01; *** = *p* < 0.001.

In parallel to the assessment of the oxidative status of the cells, cell viability was analyzed by both MTT test and propidium iodide staining in HeLa cells following 24 h exposure (Figure 2A,B).

The measurement of the metabolic activity of the cells by the MTT assay (Figure 2A) showed a dose-dependent reduction in cell viability with a maximum effect observed at 160 µM Trolox.

Moreover, as shown in Figure 2B, HeLa cells exposed for 24 h to Trolox at concentrations of 40, 80, and 160 µM showed a significant increase in the fluorescence of PI compared to control cells, while no significant change was observed at lower Trolox concentrations (Figure 2B). Propidium iodide cannot pass through a viable cell membrane, but it binds to DNA intercalating with the double helix in dead cells. Therefore, the results obtained with propidium iodide confirmed the viability results obtained with the MTT test.

### 3.2. Apoptosis Detection

**Figure 3:**
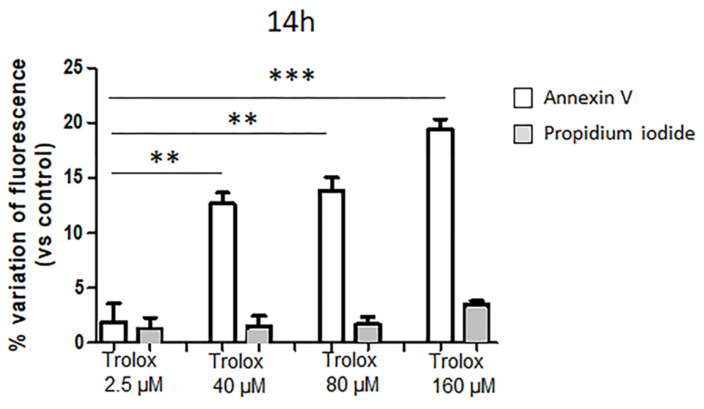
Effect of Trolox after 14 h exposure on annexin V and propidium iodide fluorescence (expressed as percentage variations of control) in HeLa cells. Details as Figure 1. Data are expressed as mean ± SEM of 3 independent experiments. Statistical significance of differences was assessed by one-way ANOVA and Dunnett test. ** = *p* < 0.01; *** = *p* < 0.001.

To deepen the knowledge about the reduced cell viability caused by the exposure to high Trolox concentrations in HeLa cells, the hypothesis of apoptosis induction was tested. One of the hallmarks of apoptosis is represented by the translocation of phosphatidylserine from the inner to the outer leaflet of the plasma membrane, thus exposing phosphatidylserine to the external cellular environment [42]. HeLa cells were exposed to 40, 80, and 160 µM Trolox, respectively, which were the concentrations found to induce oxidative stress and to decrease cell viability dose dependently, and to Trolox 2.5 µM for comparison. After 14 h exposure, the cells were double-stained with annexin V and propidium iodide, and the relative fluorescence was recorded with a multiplate reader. A shorter incubation time of 14 h was chosen in this case, after preliminary tests, in order to detect, if any, the early translocation of phosphatidylserine through annexin V binding before the occurrence of cell death. It is known that the kinetic profile of phosphatidylserine exposure in the outer layer of plasma membrane precedes the loss of membrane integrity. As previously assessed in other cell types, 14 h after the exposure to an apoptotic stimulus phosphatidylserine translocation is detectable, while the loss of membrane integrity is not yet [43]. As reported in Figure 3, the percentage variation of the annexin V fluorescence appeared strongly increased in cells exposed to 40, 80, and 160 µM Trolox for 14 h, but not in cells exposed to 2.5 µM Trolox.

### 3.3. Dose-Dependent Effect of Trolox on Basal ROS Production and Cell Volume after 2 h Exposure

After having demonstrated that Trolox can induce apoptosis at high concentrations, we tested the hypothesis of the possible involvement of the vitamin E analog in the induction of one of the first events of apoptosis, the Apoptotic Volume Decrease, which is known to occur in the first 1–2 h [31,44]. Therefore, the investigation of the dose-dependent effects of Trolox on HeLa cells was deepened at 2 h exposure.

**Figure 4:**
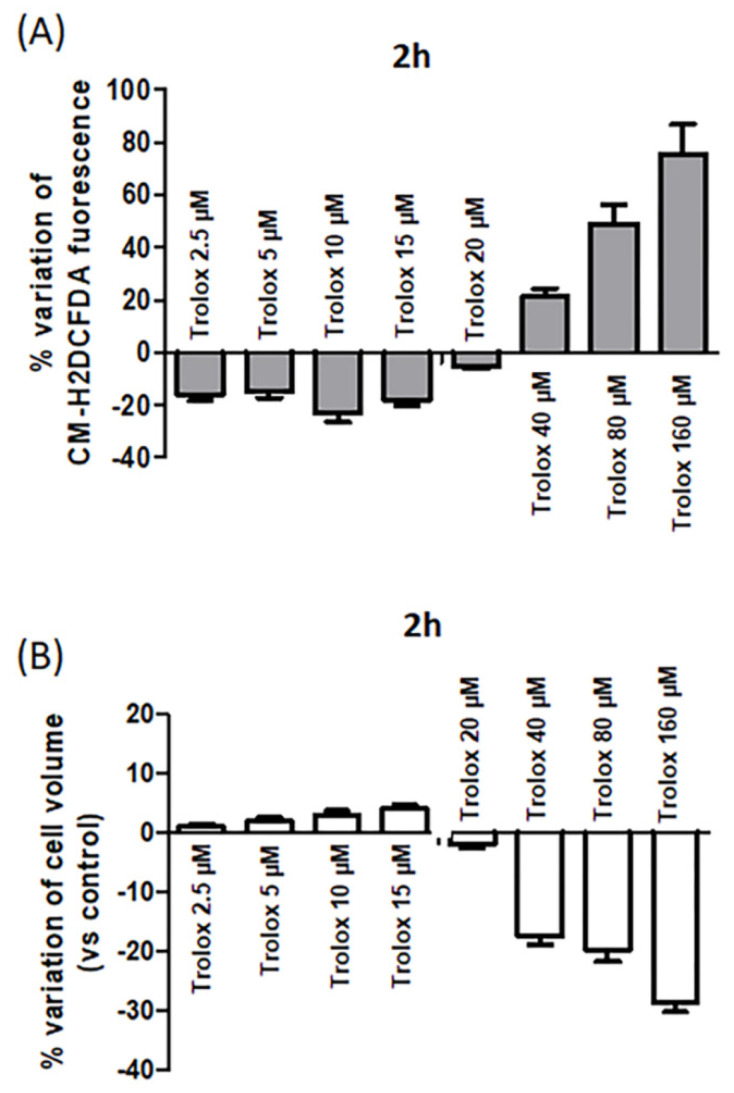
(**A**) Percentage variation of fluorescence intensity in HeLa cells exposed for 2 h to increasing concentrations of Trolox (from 2.5 to 160 µM) and then charged with CM-H_2_DCFDA. Details as Figure 1. (**B**) Effect of increasing concentrations of Trolox on cell volume after 2 h incubation. Data are expressed as cell volume percentage variation calculated as follows: (cell size of control cells − cell size of treated cells/cell size of control cells) × 100. Data are expressed as mean ± SEM of 3 independent experiments.

HeLa cells exposed for 2 h to different concentrations of Trolox (range: 2.5–160 µM) and then charged with CM-H_2_DCFDA showed the same dual behavior observed at 24 h. A decrease in the basal ROS level was evident at lower concentrations (2.5–20 µM), as indicated by the reduced fluorescence of the intracellularly trapped probe, while an increase in the basal ROS levels was detected at higher concentrations (40–160 µM) (Figure 4A).

Comparing the percentage variation of CM-H_2_DCFDA fluorescence at the highest concentrations tested after 2 and 24 h, an increased percentage variation was observed after 2 h exposure. For example, at 160 µM, the percentage variation of CM-H_2_DCFDA fluorescence was about 75% after 2 h incubation (Figure 4A), while it was about 40% after 24 h (Figure 1). It cannot be excluded that after 24 h exposure, the occurrence of cell death could cause a possible leakage of the intracellularly trapped probe from the cells, which could contribute to some extent to decrease the fluorescence signal.

In parallel, the cell size of the cells exposed to different Trolox concentrations was measured. A decrease in cell dimension at 40, 80, and 160 µM after 2 h was evident (Figure 4B), and it was indicative of isotonic cell shrinkage, namely, AVD.

### 3.4. Effect of SITS on the Trolox-Induced AVD

**Figure 5:**
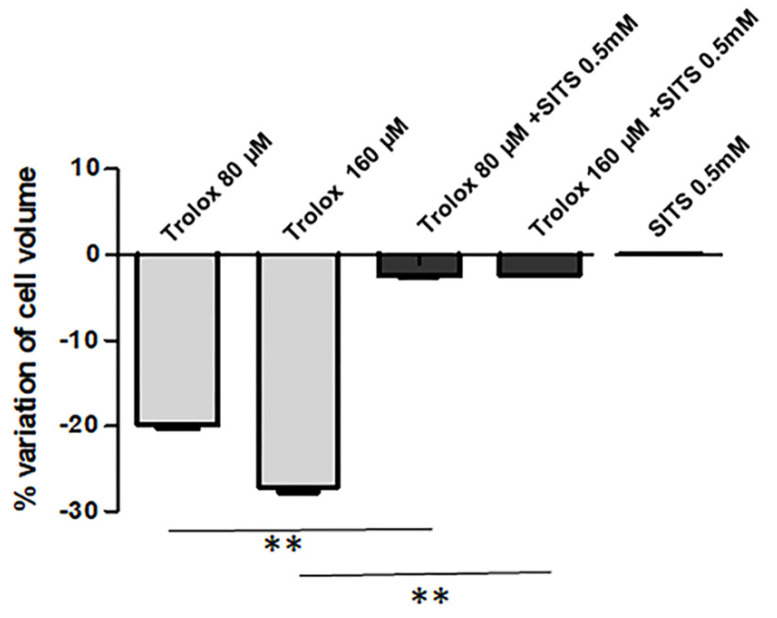
Effect of SITS (4-acetamido-4’-isothiocyanostilbene) (0.5 mM) on the Trolox-induced cell volume decrease. Details as Figure 4B. Data are reported as mean ± S.E.M of 3 independent experiments. Statistical significance was assessed by one-way ANOVA and Dunnett test. ** = *p* < 0.01.

AVD is known to be due to the loss of K^+^ and Cl^−^ from the cells [45]. As reported in Figure 5, when HeLa cells were preincubated with the 0.5 mM disulfonic stilbene derivative SITS (4-Acetamido-4′-isothiocyanato-stilbene-2,2′-disulfonic acid), a known inhibitor of Cl^−^ channels [46], and then exposed to Trolox 80 and 160 µM for 2 h, the Trolox-induced isotonic shrinkage was completely prevented. On the other hand, SITS alone was not able to produce any significant alteration of cell size.

## 4. Discussion

Oxidative stress, which occurs when the production and accumulation of reactive species in cells and tissues are not balanced by the antioxidant defenses of a biological system, has been recognized as an important factor in the genesis of several diseases, including chronic and degenerative diseases [47]. This accounts for the great interest devoted in the last years to antioxidant compounds and to their capacity in protecting cells from oxidative damage. However, some studies have pointed out that antioxidants can exert prooxidant effects under particular conditions, depending on their concentration and the nature of surrounding molecules [48]. This study is aimed to analyze the dose-dependent effect of Trolox, a widely utilized synthetic analog of vitamin E, on the basal ROS production and viability of HeLa cells, focusing on the ability of the compound to induce apoptosis at high concentrations.

In HeLa cells, Trolox showed significant antioxidant activity, expressed as the ability to reduce the endogenous ROS production detected by the ROS-sensitive probe CM-H_2_DCFDA. The antioxidant activity was expressed at concentrations included in the range of 2.5–15 µM. At 20 µM, the Trolox antioxidant activity appeared reduced compared to the previous concentrations, as observed after 2 h incubation, or disappeared, as observed after 24 h incubation. At 40 µM, Trolox activity was reversed becoming prooxidant. The prooxidant effect showed a marked concentration-dependence with the highest effect observed at the highest concentration tested (160 µM). The prooxidant effect was accompanied by a reduction in cell viability as assessed by MTT test and propidium iodide staining.

These results are in agreement with results obtained on HUVEC cells exposed for 24 h to a wide range of Trolox concentrations [39]. However, in the case of HeLa cells, the prooxidant effects appeared at lower concentrations compared to HUVEC cells, where the prooxidant behavior, accompanied by a reduction in cell viability, was detected at much higher concentrations (500–1000 µg/mL) [39]. This points out the cell-type specificity in the dual behavior of Trolox and in the threshold concentration for the inversion of its antioxidant behavior in prooxidant behavior. The cell-type specificity could be influenced by the oxidative status of the cell and by the intracellular antioxidant defenses. The appearance of the prooxidant behavior as a consequence of the accumulations of phenoxyl radical (PhO·), arising, in turn, from the antioxidant activity of Trolox, can depend on the intracellular concentrations of antioxidants, such as GSH or ascorbic acid, able to neutralize the radical forms of Trolox. In our experimental model, 40 µM seems to be the threshold concentration for the inversion.

In our study, the reduction in the cell viability was ascribed to apoptosis, as assessed by the early detection (after 14 h incubation) of phosphatidylserine translocation by annexin V binding.

The study on the prooxidant effect of Trolox in HeLa cells was deepened by the analysis of the early effects of the vitamin E analog after 2 h incubation. The dual behavior, antioxidant at lower concentrations and prooxidant at higher concentrations, was evident already after 2 h incubation, with a pronounced increase in the intracellular oxidative stress at 80 and 160 µM. At this early stage, the prooxidant effect of Trolox was paralleled by the isotonic shrinkage of the cells, ascribed to AVD. This represents one of the first events of apoptosis, known to start within 0.5–2 h after apoptosis induction [37]. AVD is mainly due to KCl release from the cells, followed by osmotic loss of water. KCl occurs thanks to the activation of K^+^ and Cl^−^ channels in several cell types [31,32,33,49]. The Cl^−^ channel involved in this process is known to be the volume-sensitive outwardly rectifying (VSOR) anion channel in different cell types [31,50,51]. They are known to be activated by osmotic cell swelling and to be involved in cell volume regulation and apoptotic cell death. VSOR channels are expressed in several cell types including HeLa cells [52,53].

In our study, we tested the hypothesis of possible induction of these channels by exposure to high concentrations of Trolox, 80 and 160 µM, respectively, which are the concentrations able to exert a marked prooxidant effect in HeLa cells. The use of SITS, known to block Cl^−^ channels including VSOR channel [54], was able to completely inhibit the Trolox-induced isotonic cell shrinkage, demonstrating the role of the vitamin E analog in the induction of volume-sensitive Cl^−^ channels, and, in turn, in AVD induction. On the other hand, the only treatment with SITS did not produce any alterations in cell volume, suggesting that these channels are normally closed in basal conditions.

As regards the mechanism through which Trolox can activate volume-sensitive Cl^−^ channels in HeLa cells, VSOR have been demonstrated to be sensitive to ROS in HeLa cells [48]. The obtained results demonstrate that Trolox at high concentrations can increase the intracellular ROS basal level. This, in turn, could activate volume-sensitive Cl^−^ channels, which play a key role in the apoptotic process. As demonstrated in HeLa, U937, and PC12 cells [31,45,53], the activation of AVD-inducing VSOR Cl^−^ channel is an early requisite event of apoptosis, because its inhibition can prevent further apoptotic events such as caspase-3 activation, cytochrome *c* release, and DNA laddering. Therefore, this study, for the first time, disclosed the role of Trolox at high concentrations in AVD and VSOR Cl^−^ channel activation, although future studies will be needed to analyze the effect of high Trolox concentrations on AVD induction in comparison with other oxidant agents and to deepen the mechanisms and signaling events underlying the Trolox effect.

## 5. Conclusions

In conclusion, this study clarified the concentration dependence of the antioxidant and prooxidant activity of Trolox in HeLa cells over a wide range of concentrations, disclosing the threshold inversion concentration of its antioxidant/prooxidant behavior. Although this study has been carried out on a single cellular type, the obtained results can be used as a model for further studies on other antioxidants in other cell types. In fact, this study underlines the need to evaluate in advance the dose dependence of the effects of the substance under investigation, its possible prooxidant behavior, and the antioxidant/prooxidant inversion threshold before carrying out any evaluation on its biological effect on any experimental system.

Moreover, this study disclosed for the first time the role of the vitamin E analog at high concentrations in the induction of one of the first events of apoptosis, AVD, through the induction of oxidative stress and VSOR Cl^−^ channel activation.
